# Molecular mechanism of regulation of OGG1: tuberin deficiency results in cytoplasmic redistribution of transcriptional factor NF-YA

**DOI:** 10.1186/1750-2187-4-8

**Published:** 2009-12-29

**Authors:** Samy L Habib

**Affiliations:** 1 South Texas Veterans Healthcare System, Geriatric Research, Education and Clinical Center, Department of Medicine, University of Texas Health Science Center, San Antonio, Texas 78229, USA

## Abstract

The tuberous sclerosis complex (TSC) is caused by defects in one of two tumor suppressor genes, TSC-1 or TSC-2. TSC-2 gene encodes tuberin, a protein involved in the pathogenesis of kidney tumors, both angiomyolipomas and renal cell carcinomas. On the other hand, mice-deficient in the DNA repair enzyme OGG1 spontaneously develop adenoma and carcinoma. Downregulation of tuberin results in a marked decrease of OGG1 and accumulation of oxidative DNA damage, (8-oxodG) in cultured cells. In addition, tuberin haploinsufficiency is associated with the loss of OGG1 and accumulation of 8-oxodG in rat kidney tumor. Deficiency in tuberin results in decreased OGG1 and NF-YA protein expression and increased 8-oxodG in kidney tumor from TSC patients. In the current study, molecular mechanisms by which tuberin regulates OGG1 were explored. The deficiency of tuberin was associated with a significant decrease in NF-YA and loss of OGG1 in kidney tumors of Eker rat. Downregulation of tuberin by siRNA resulted in a marked decrease in NF-YA and OGG1 protein expression in human renal epithelial cells. Localization of NF-YA in wild type and tuberin-deficient cells was examined by western blot and immunostaining assays. In wild type cells, NF-YA was detected in the nucleus while in tuberin deficient cells in the cyotoplasm. Introducing adenovirus-expressing tuberin (Ad-TSC2) into tuberin-deficient cells restored the nuclear localization of NF-YA. These data define a novel mechanism of regulation of OGG1 through tuberin. This mechanism may be important in the pathogenesis of kidney tumors in patients with TSC disease.

## Findings

8-Oxo-deoxyguanine (8-oxo-dG) is a major form of oxidative DNA damage. 8-Oxo-dG has been implicated in carcinogenesis, ageing and several age-related degenerative diseases [1-3]. 8-Oxo-dG is repaired primarily via the DNA base excision repair pathway. The gene coding for the DNA repair enzyme that recognizes and excises 8-oxo-dG is 8-oxoG-DNA glycosylase (OGG1) [3,4]. Deficiency in OGG1 has important functional consequences, and compromises the ability of cells to repair DNA [4]. In addition, OGG1 deficiency in yeast, as well as formamidopyrimidine-DNA glycosylase (FPG) deficiency in bacteria, results in a spontaneous mutator phenotype [5]. However, increasing impairment in DNA repair can contribute to the genomic instability and in consequence to cancer [6]. The steady-state levels of 8-oxo-dG, which reflect the balance between its continuous generation and removal, are significantly higher in livers of *OGG1*^-/- ^mice compared to wild-type animals [6]. The *OGG1 *gene is somatically mutated in some cancer cells and is highly polymorphic among humans [7,8]. Moreover, loss of heterozygosity at the *OGG1 *allele is found in 85% of 99 human kidney clear cell carcinoma samples, identifying that loss of OGG1 function as a possible consequence of multistep carcinogenesis in the kidney [8]. Nuclear factor-YA (NF-YA) has been identified as a transcription factor that binds to a consensus sequence in the OGG1 promoter [9]. NF-Y is a ubiquitous that specifically recognizes a CCAAT box motif and regulates *hOGG1 *expression as well as genes that regulate development and cell cycle [9].

The *TSC2 *gene encodes the protein tuberin [10]. Tuberin is a structurally complex protein containing several functional domains [11]. Tuberin is normally exists in an active state and forms a heterodimeric complex with hamartin, the protein encoded by the *TSC1 *gene. Tuberin can be inactivated by several mechanisms including changes in subcellular localization, dissociation from hamartin and other regulatory proteins, or degradation of the hamartin-tuberin complex [12]. Deficiency or inactivation of tuberin is associated with human malignancies including RCC [13].

The constitutive expression of OGG1 in heterozygous Eker rat (TSC2^+/-^) kidneys is lower than in wild type rats suggesting that these proteins may be functionally linked [14,15]. In addition, downregulation of tuberin results in a marked decrease in the abundance of OGG1 in human renal epithelial cells [16]. Moreover, mouse embryonic fibroblasts deficient in tuberin (*TSC2*^-/- ^and *TSC2*^+/-^) also express very low levels of OGG1 mRNA and protein and undetectable level of OGG1 activity accompanied by accumulation of 8-oxodG [16]. The decrease in OGG1 mRNA in tuberin-deficient cells suggests that decreased transcription is one potential mechanism responsible for down regulation of OGG1 protein [16]. In addition, tuberin deficiency is associated with downregulation of protein and mRNA expression of OGG1 as well as NF-YA expression and accumulation of 8-oxodG in angiomyolipoma kidney tissue of TSC patients [17]. The present study was conducted to investigate the molecular mechanism of regulation of OGG1 in cell culture model.

**Figure 1:**
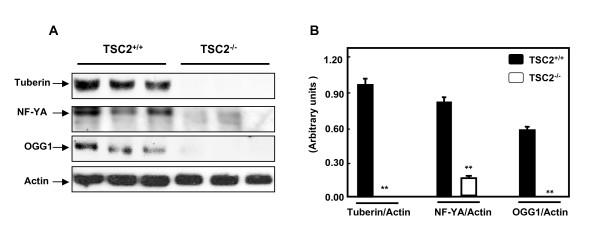
**Deficiency in tuberin is associated with significant decrease in NF-YA and loss of OGG1 expression in kidney tumor of Eker rats**. **A**. Immunoblot analysis of tuberin, NF-YA and OGG1 protein expression in normal kidney of wild type rats and tumor kidney tissue from Eker rats. Actin was used as loading control. **B**. Histograms represent means ± SE (n = 3). Significant difference from wild type rat is indicated by ** *P *< 0.01.

To determine the effect of tuberin on expression of NF-YA, kidney from wild type rats and tumor kidney tissue from Eker rats were examined by western blot analysis. Loss of tuberin was associated with loss of OGG1 and significant decrease in NF-YA in tumor kidney tissue of Eker rats (Fig. 1). These data suggest that tuberin is an important tumor suppressor protein involve in the regulation of OGG1 abundance through NF-YA.

**Figure 2:**
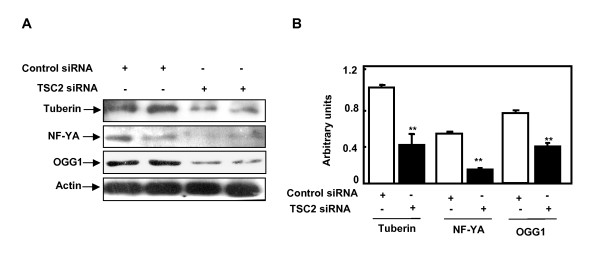
**Downregulation of tuberin expression in human renal epithelial cells results in decrease in NF-YA and OGG1 expression**. **A**. Immunoblot analysis of tuberin, NF-YA and OGG1 in HEK 293 cells transfected with siRNA directed against *TSC2 *for 48 h. Actin was used as a loading control. **B**. Histograms represent means ± SE (n = 2). Significant differences from cells transfected with the TSC2-specific siRNA are indicated by **P < 0.01.

To explore the role of tuberin in the regulation of OGG1 expression, tuberin was first downregulated using specific siRNA against *TSC2 *gene in human renal epithelial cells. The cells transfected with the duplex siRNA oligonucleotide complementary to *TSC2 *had decreased tuberin protein expression compared to cells transfected with scrambled control oligonucleotides (Fig. 2). Downregulation of tuberin resulted in a decrease of NF-YA and OGG1 protein expression (Fig. 2).

**Figure 3:**
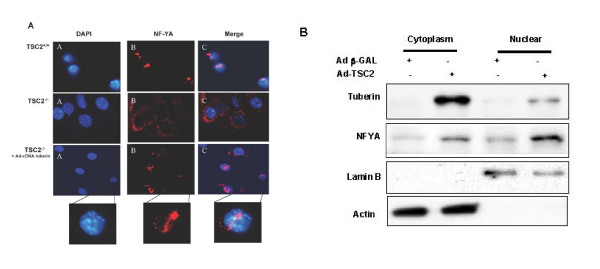
**Mislocalization of NF-YA in tuberin-deficient cells**. **A**. *TSC*2^-/- ^and *TSC*2^+/+ ^cells were plated on a 2-well chamber slide. Cells were fixed then immunostained with primary anti-NF-YA antibody and secondary anti-goat IgG labeled with Alexa Fluor 488 (red fluorescence). The same cells were also incubated with DAPI (blue fluorescence, same microscopic fields) to identify nuclei. Overlay of NF-YA and DNA staining, demonstrating nuclear localization of NF-YA in the wild type and tuberin-deficient cells infected with adenovirus expressing tuberin. **B**. Nuclear and cytoplasmic fraction proteins were extracted from *Tsc2*^-/- ^cells non-infected and infected with adenovirus expressing tuberin. Immunoblot analysis of tuberin and NF-YA in the nuclear and cytoplasmic fractions was analyzed as described in Method section. Lamin B and actin antibodies were used as nuclear and cytoplasmic markers, respectively.

We next examined whether tuberin deficiency influences the subcellular localization of NF-YA, which must as a transcription factor localize in the nucleus. We examined the localization of NF-YA in wild type and in tuberin-null cells by immunofluorescence staining. In wild type cells, NF-YA staining was detected primarily in the nucleus (Fig. 3A), while in tuberin-null cells NF-YA was seen only in perinuclear cytoplasm (Fig. 3A). Infection of tuberin-null cells with Ad-TSC2 restored the wild type pattern of predominantly nuclear NF-YA (Fig. 3A).

To confirm that tuberin-deficiency results in cytoplasmic redistribution of NF-YA, cyoplasmic and nuclear fractionation was performed in tuberin-null and in tuberin-null cells infected with Ad-TSC2. Data show that NF-YA localized in the cyoplasmic fraction of the tuberin-deficient cells (Fig. 3B). Introduction of tuberin into tuberin-null cells using Ad-TSC2 significantly increased the nuclear localization of NF-YA (Fig. 3B). These data suggest that tuberin is a key molecule involve in the regulation of OGG1 function through distribution of NF-YA between nucleus and cytoplasm.

In summary, tuberin deficiency in tumor kidney tissue of Eker rat is associated with decreased in NF-YA and OGG1 expression. Downregulation of tuberin in renal cells results in decreased NF-YA and OGG1 expression. The major of interest in this study is that tuberin deficiency is associated with the localization of NF-YA to the cytoplasm rather than nucleus thus providing a mechanism for the decreased transcription of OGG1 observed in tuberin-deficient cells. Localization of NF-YA to the cytoplasm would abrogate its function as a transcription factor. These data suggest that tuberin plays a major role in protecting the cells from the oxidative DNA damage by regulating localization of NF-YA, the major transcription factor regulating OGG1 gene. Further studies to identify the mechanisms by which tuberin deficiency regulates localization of NF-YA should help clarify how tuberin regulates DNA repair pathways involved in tumor formation.

## Materials

### Animals

Wild type (*TSC-2*^+/+^) and Eker male rats (mutant *TSC-2*^+/-^) were purchased from a breeding colony maintained at the University of Texas MD Anderson Cancer Center, Smithville, TX. The animals were allowed food and water *ad libitum *throughout the experiments. Animals were euthanized at 12 months for nephrectomy. The kidneys were dissected and used for biochemical assays.

### Cell culture

Mouse embryonic fibroblasts (MEFs) derived from *Tsc2*^-/-^, and *Tsc2*^+/+ ^embryos were generously provided by Dr. D. J. Kwiatkowski (Harvard Medical School, MA). The cells were grown in DMEM supplemented with 10% fetal bovine serum (FBS). Human kidney epithelial cells (HEK 293) were obtained from American Type Culture Collection (Manassas, VA) and maintained in DMEM with 10% FBS. All cell lines were grown at 37°C in a humidified atmosphere of 5% CO_2_.

### Downregulation of tuberin by siRNA

Downregulation of tuberin in HEK293 cells was performed as previously described [16]. SMART selected siRNA duplexes with "UU" overhangs and 5' phosphate on the antisense strand were obtained in a kit from Dharmacon/Upstate, NY. The siRNA specific for *TSC2 *was a mixture of 4-pooled duplexes. According to the manufacturer, these siRNA efficiently blocks tuberin expression by 70%. Forty-eight hours after transfection, cells were harvested for Western blot analysis. The control construct used in parallel experiments contains 4 pooled, non-specific siRNA duplexes provided by Dharmacon/Upstate.

### Adenovirus Infection

Tuberin null cells grown on a 2-well chamber slide or in 6 well plates were infected with a recombinant adenovirus expressing tuberin (Ad-TSC2). An adenovirus expressing protein (Ad β-GAL) was used as a control. The cells were grown to 60-70% confluency in complete medium. The cells were infected at 20 multiplicity of infection (MOI). Forty-eight hours after infection, cells were harvested for either western blot or immunostaining assay.

### Detection of NF-YA localization

Wild type and tuberin null cells were grown on a 2-well chamber slide (Becton Dickinson, MA) for 24 h. Cells were washed 3× with PBS then fixed with 4% parafomaldehyde for 30 min followed by 0.4% Triton X-100 for 10 min at RT. Cells were subsequently washed with PBS and incubated with PBS containing 3% BSA at RT for 1 h in a humidified atmosphere. Cells were then washed with PBS and incubated with anti-NF-YA antibody (1: 200 dilution) at RT for 1 h, then washed with PBS. Cells were incubated with anti-rabbit secondary antibody labeled with Alexa Fluor 488 and FITC (1:200 dilution) for 15 min. Cellular DNA was stained with DAPI containing gold antifade mount. Cells were visualized by confocal fluorescence microscopy.

### Cell lysates fractionation

Cytoplasmic and nuclear fractions were extracted from the cell lysates using nuclear and cytoplasmic fractionation kit (Pierce, IL).

### Protein extraction and immunoblot analysis

Cell lysates and kidney cortex tissue homogenates were prepared as previously described [16,17]. Protein concentration was determined with the Bradford assay [18] using bovine serum albumin as a standard. Western blot analysis was performed as previously described [17]. Rabbit polyclonal antibody raised against human OGG1 protein was generously provided by Dr. S. Mitra (University of Texas Medical Branch at Galveston, Texas). Goat anti-NF-YA, rabbit anti-tuberin and rabbit anti-lamin B antibodies were purchased from Santa Cruz Biotechnology and mouse β-actin antibody from Oncogene Research Products. Expression of each protein was quantified by densitometry using National Institutes of Health Image 1.62 software.

### Statistics

Data are presented as mean ± standard error. Statistical differences were determined using ANOVA followed by Student Dunnett's (Exp. vs. Control) test using 1 trial analysis. *P*-values less than 0.05 were considered statistically significant.

## Abbreviations

TSC2: tuberous sclerosis complex-2; OGG1: 8-oxoG-DNA glycosylase; RCC: renal cell carcinoma.

## Competing interests

The author declares that he has no competing interests.

## Authors' contributions

SLH conceived the concept, designed the study, performed the cell culture and animals experiments, Western and immunohistochemistry assays and prepared the manuscript.
